# Integrated behavioural and stable isotope data reveal altered diet linked to low breeding success in urban-dwelling blue tits (*Cyanistes caeruleus*)

**DOI:** 10.1038/s41598-017-04575-y

**Published:** 2017-07-10

**Authors:** Christopher J. Pollock, Pablo Capilla-Lasheras, Rona A. R. McGill, Barbara Helm, Davide M. Dominoni

**Affiliations:** 1 Institute of Biodiversity, Animal Health and Comparative Medicine, University of Glasgow, Glasgow, G128QQ UK; 2 Centre for Ecology and Conservation, College of Life and Environmental Sciences, University of Exeter, Penryn Campus, Cornwall, TR10 9EZ UK; 3 NERC Life Sciences Mass Spectrometry Facility, Scottish Universities Environmental Research Centre (SUERC), East Kilbride, G750QF UK; 4 Department of Animal Ecology, Netherlands Institute of Ecology, Wageningen, 6708 PB The Netherlands

## Abstract

Animals often show reduced reproductive success in urban compared to adjacent natural areas. The lower availability and quality of natural food in cities is suggested as one key limiting factor. However, only few studies have provided conclusive support by simultaneously assessing food availability, diet and fitness. We consolidate this evidence by taking a holistic approach, comparing blue tits breeding in forest, suburban and urban areas. We (a) assessed arthropod availability, (b) investigated parental provisioning behaviour, (c) inferred diet through stable isotope analysis, and (d) measured reproductive success. At the urban site, we found a significant reduction in caterpillar availability, the main food source of blue tits, and consequently urban tits fed their offspring with fewer caterpillars than forest and suburban birds. Stable isotope analysis confirmed that diet in the urban area was fundamentally different than in the other sites. Reproductive success was lower in both urban and suburban sites compared to the forest site, and was positively associated with volume of provisioned caterpillars. Our findings provide strong integrative evidence that urban blue tit nestlings are not receiving a suitable diet, and this may be an important limiting factor for urban populations of this and potentially many other species.

## Introduction

Urbanization is a rapidly growing global phenomenon that is associated with increased environmental pollution, spread of novel diseases, and loss of biodiversity and ecosystem services^1, 2^. While a selected few species thrive in cities, many others will experience a mix of benefits and costs of urban life. Among wild species that establish themselves in urban areas, changes in physiology and behaviour can be observed^3^, and urban populations often exhibit decreased health and reproductive success compared to counterparts in more natural habitats. In birds, clutch size, fledging success and nestling body mass are generally lower in urban than in rural populations^4–6^. Similar patterns occur also in mammals: coyotes living within human settlements were lighter and more prone to infections compared to those in natural forests^7^. However, cities also provide advantages, such as high availability of nesting sites^8^ and additional food sources^9^ that may in some cases improve health and survival^10^, or at least buffer against negative health effects of urbanisation^11^. If mismatches occur between an animal’s perception of attractive urban features and the costs they impose on fitness, cities could become ecological traps for wild animals^8, 12^.

Such mismatches can be driven by a disconnection between quantity and quality of resources, for example by food that is readily available but of lower quality. Replacing specialised, high-quality food with a broad, lower-quality diet can be particularly dangerous if it selectively impairs sensitive life-cycle phases, especially early life growth^8, 13, 14^. A seasonal shift in the balance between benefits and costs of urban life may occur in various songbird species, which are drawn to cities by anthropogenic foods (directly or indirectly supplemented) to enhance winter survival^15^, but whose offspring depend on highly specialised insect food^16, 17^. Benefits of provisioning birds in winter have been at least partly confirmed^18^, including positive increases in lay date, clutch size, and fledgling success (reviewed in refs 18, 19). However, recent studies have also highlighted potential negative consequences for breeding success^20–22^. For example, during the breeding period several studies support the idea that the urban habitat does not provide nestlings with an adequate diet, both in terms of quantity and quality of available resources. Gladalski and collaborators showed that caterpillar availability was scarcer in an urban park than a rural area, and suggest it is a contributor to lower clutch size for urban blue tits (*Cyanistes caeruleus*)^23^. Data on stress physiology and a food-supplementing experiment showed that the urban diet was nutritionally insufficient for nestling American Crows (*Corvus brachyrhynchos*)^24^, while Seress *et al*.^25^ as well as Isaksson and Andersson^26^ found low quality of nestling diet in urban house sparrows (*Passer domesticus*) and great tits (*Parus major*), respectively. Overall, current evidence suggests an association between potentially compromised nestling diets in the city and poor reproductive success^6, 27^. However, most of these studies focused on one or at most two aspects of this puzzle. For instance, they assessed food availability and breeding success, but not dietary intake^23^, or used food manipulation experiments to demonstrate the reproductive consequences of poor diet, but did not examine whether the availability of preferred diet is actually lower in urban than forest habitats^24^.

Filling the gap of simultaneous analysis of food availability, provisioning, and breeding success is necessary to understand the extent to which changes in food availability may impact health and fitness in urban areas^7^. In this study we therefore employ an integrated and holistic approach by examining what type of food is available in urban and forest environments, what diet the birds in these different habitats provision, and what effect an altered urban diet has on reproductive success. Naturally, such a comprehensive analysis is focused on few field sites, trading off greater depth against a greater number of study sites. Hence, we intend for this study to provide tentative insights into processes that reduce reproductive performance of urban birds, to be then tested at additional locations.

We use the blue tit as study species, because nestling fitness is known to be tightly linked to a highly specialised food source, caterpillars^28, 29^. In this species, the performance of parents directly affects the prospects of their remarkably large clutch sizes^28^. Blue tits therefore attempt to synchronize the peak energy demands of their offspring to the peak abundance of caterpillars^30^, and in poor habitats, mismatches can have severe fitness consequences^31^. Blue tits are successful urban colonizers, but in cities, where caterpillar availability is likely poor due to low tree density^26^, they show reduced clutch size, nestling mass and fledging success compared to forest sites^4, 6^. To achieve our aim, we compared food availability, provisioning behaviour, and reproductive success of blue tits in an urban, suburban and forest site (Table S1) during the breeding season. Additionally, we used stable isotope analysis to infer dietary niche, revealing what food sources were taken up in the bodies of blue tits^32^.

Specifically, we tested the hypothesis that a combination of shortage of optimal nestling food and parental provisioning of non-optimal diets could reduce reproductive success in urban areas. First, we sampled arthropods at regular intervals during the tits’ breeding season and predicted lower caterpillar availability in urban environments. Second, using infrared cameras inside nest-boxes, we recorded parental provisioning behaviour to characterize the type and size of food items fed to the nestlings. We predicted that urban blue tit parents will feed other dietary sources, including anthropogenic items, to compensate for the lack of caterpillars. Third, we assessed how much of this diet was assimilated into tissues by means of stable isotope analysis. Fourth, we related the diet fed to offspring to the breeding success of pairs at our different sites, and predicted that low number of dietary caterpillars would be negatively correlated to breeding success.

Quantifying dietary information by stable isotope analysis is a powerful approach in ecology^32^, as it enables detection of trophic levels, habitat-specific dietary sources and consumption of supplements^18, 33^. These values are applicable from various avian tissues, including eggs and blood^34, 35^. Commonly, nitrogen (δ^15^N) and carbon (δ^13^C) stable isotopes are applied to infer trophic level and to trace carbon sources. Nitrogen sources are useful to assess the trophic level, which is inferred from the ratio of the heavier to lighter N stable isotope value in an animal’s tissues, with animals that occupy higher trophic levels (like predatory invertebrates) showing higher δ^15^N values. We predicted urban tits to show higher δ^15^N values due to food in bird feeders being rich in this isotope, but possibly also through an increased uptake of predatory arthropods which constitute a good portion of blue tits’ diet^36^, to compensate for the expected lack of caterpillars. Carbon sources allow one to distinguish the base of a food web because the isotopic signatures reflect metabolic differences in photosynthetic pathways of primary producers such as C_3_ (eg, all woody trees, many grasses) and C_4_ plants (eg, maize, sugar), with enriched δ^13^C in C_4_ plants. Previous studies indicated higher δ^13^C values in urban animals compared to rural counterparts^7, 9^, likely due to consumption of anthropogenic food which is rich in C_4_-derived foods^9^, but also to habitat differences in plant density and composition^37^. Thus, we predicted higher δ^13^C signatures at our more urban site. In addition, if urban tits integrate their diet from other sources, then the isotopic “niche” should be larger compared to that of forest conspecifics. Last, we also obtained isotope samples from adult blood and egg yolk to assess (a) whether adult signatures differ from nestling as parent blue tits are known to selectively feed nestlings with caterpillars while their own diet might be broader^28^ and (b) whether such differences may be partly carried over to nestlings. If mothers still fed on broader food sources during egg formation, their eggs should show isotope values that are similar to those of adults.

## Results

### Reproductive performance and body mass

Lay date, clutch size and hatch date did not significantly change across sites (Tables 1, 2 and 3; Fig. S1). Hatching success was significantly affected by site (P = 0.018), and post hoc tests revealed that this was mainly due to hatching success being significantly higher in the suburban compared to the urban site. Fledging success was strongly affected by site (P < 0.001, Table 3, Fig. S1), and so was total number of fledglings per brood (P < 0.001; site-specific estimates: forest = 5.06; suburban = 2.63; urban = 0.38). Post hoc tests showed that both parameters strongly decreased with increasing urbanisation, and all pairwise differences were significant (Table 1).

**Table 1:** Summary of Tukey’s posthoc tests for all linear contrasts of all models.

Analysis	Response variable	Type of model	Contrasts (Tukey’s post hoc tests)								
			Forest-suburban			Forest-urban			Urban-suburban		
			Estimate	Std. error	P value	Estimate	Std. error	P value	Estimate	Std. error	P value
Life history and body mass	Lay date	Poisson GLM	−0.13	0.05	**0.018**	−0.08	0.05	0.205	0.05	0.05	0.571
	Clutch size	Poisson GLM	−0.10	0.09	0.488	−0.13	0.09	0.291	−0.03	0.10	0.946
	Hatch date	Poisson GLM	−0.07	0.04	0.133	−0.04	0.04	0.503	0.03	0.04	0.671
	Hatching success	Binomial GLM	0.97	0.46	0.088	−0.48	0.43	0.502	−1.45	0.35	**<0.001**
	Fledging success	Binomial GLM	−7.13	1.87	**<0.001**	−12.50	2.25	**<0.001**	−5.38	1.13	**<0.001**
	Number of fledglings	Poisson GLM	−0.65	0.14	**<0.001**	−2.59	0.31	**<0.001**	−1.94	0.32	**<0.001**
	Nestling body mass at day 13	LMM	−1.69	0.32	**<0.001**	−1.37	0.41	**0.002**	0.32	0.45	0.755
	Adult body mass	LMM	−0.25	0.55	0.891	0.12	0.46	0.965	0.37	0.56	0.789
Caterpillars	Total weight (log)	LMM				−0.01	0.003	**<0.001**			
	Total numbers (log)	LMM				−0.60	0.08	**<0.001**			
Provisioning behaviour	Visits per nestling (log)	LMM	0.04	0.21	1.000	0.49	0.16	**0.007**	0.45	0.21	0.098
	Total visits per nest	LMM	−6.59	3.98	0.292	−6.57	3.06	0.096	0.03	3.98	1.000
	Caterpillar volume (log)	LMM	−0.33	0.36	0.631	−0.66	0.28	**0.049**	−0.33	0.36	0.639
	Number of caterpillars	LMM	−9.49	2.78	0.002	−13.02	2.14	**<0.001**	−3.52	2.78	0.411
	Number of other arthropods (log)	LMM	0.10	0.41	0.967	0.42	0.31	0.344	0.32	0.41	0.707
	Number of unidentified items	LMM	2.02	1.30	0.264	3.94	0.96	**<0.001**	1.92	1.31	0.304
Stable isotopes	Eggs δ^13^C	LM				1.11	0.47	**0.031**			
	Eggs δ^15^N	LM				−0.47	0.22	**0.050**			
	Adults δ^13^C	LM	0.66	0.17	**0.001**	0.71	0.15	**<0.001**	0.05	0.18	0.959
	Adults δ^15^N	LM	−0.47	0.36	0.387	0.23	0.31	0.734	0.71	0.38	0.161
	Nestlings δ^13^C	LM	0.74	0.23	**0.009**	0.76	0.22	**0.005**	0.02	0.26	0.997
	Nestlings δ^15^N	LM	−0.18	0.28	0.801	1.19	0.27	**<0.001**	1.37	0.32	**0.001**

For full model results see Tables 3, 4, 5 and 6. Bold characters highlight significant differences.

**Table 2:** Estimated least square means obtained from model predictions (full models given in Tables 3, 4, 5 and 6).

Analysis	Response variable	Type of model	Estimated means					
			Forest		Suburban		Urban	
			Mean	Std. error	Mean	Std. error	Mean	Std. error
Life history and body mass	Lay date	Poisson GLM	3.45 (31.46)	0.03	3.32 (27.67)	0.04	3.37 (29.1)	0.03
	Clutch size	Poisson GLM	2.15 (8.59)	0.06	2.05 (7.75)	0.07	2.02 (7.52)	0.07
	Hatch date	Poisson GLM	4.03 (56.41)	0.02	3.96 (52.55)	0.03	3.99 (54.23)	0.03
	Hatching success	Binomial GLM	1.15 (0.76)	0.32	2.12 (0.89)	0.34	0.67 (0.66)	0.30
	Fledging success	Binomial GLM	5.98 (0.99)	1.26	−1.15 (0.24)	1.05	−6.52 (0.01)	1.31
	Number of fledglings	Poisson GLM	1.62 (5.06)	0.08	0.97 (2.63)	0.12	−0.97 (0.38)	0.30
	Nestling body mass at day 13	LMM	11.36	0.18	9.67	0.26	9.98	0.37
	Adult body mass	LMM	11.39	0.32	11.14	0.45	11.51	0.33
Caterpillars	Total weight	LMM	0.013	0.003			0.001	0.003
	Total numbers (log)	LMM	0.65 (1.92)	0.09			0.06 (1.06)	0.09
Provisioning behaviour	Visits per nestling (log)	LMM	1.33 (3.74)	0.11	1.36 (3.91)	0.18	1.81 (6.10)	0.11
	Total visits per nest	LMM	20.22	2.16	13.62	3.34	13.65	2.17
	Caterpillar volume (log)	LMM	4.90 (134.12)	0.20	4.57 (96.34)	0.30	4.24 (69.49)	0.20
	Number of caterpillars	LMM	16.96	1.51	7.47	2.33	3.94	1.51
	Number of other arthropods (log)	LMM	0.86 (2.37)	0.22	0.97 (2.63)	0.35	1.29 (3.63)	0.22
	Number of unidentified items	LMM	1.66	0.68	3.68	1.12	5.60	0.69
Stable isotopes	Eggs δ^13^C	LM	−23.70	0.33			−22.59	0.33
	Eggs δ^15^N	LM	5.32	0.16			4.84	0.16
	Adults δ^13^C	LM	−25.60	0.10	−24.94	0.14	−24.89	0.11
	Adults δ^15^N	LM	5.86	0.20	5.39	0.29	6.09	0.23
	Nestlings δ^13^C	LM	−26.60	0.13	−25.85	0.19	−25.83	0.17
	Nestlings δ^15^N	LM	5.61	0.16	5.43	0.23	6.81	0.21

For GLMs as well as LMMs in which the response variable was log-transformed, we give both the estimated mean and in brackets the back-transformed mean. The standard errors of those means are not back-transformed.

**Table 3:** Results from models (after backward selection) investigating how life history traits and body mass varied across the three study sites.

Response variable				
Explanatory variable	Estimate	Std. Error	z value	P value
(a) Lay date (poisson GLM)				
Intercept	3.47	0.05	72.17	<0.001
(b) Clutch size (poisson GLM)				
Intercept	2.59	0.18	14.17	<0.001
Lay date	−0.01	0.01	−2.33	**0.020**
(c) Hatch date (poisson GLM)				
Intercept	3.70	0.08	44.43	<0.001
Lay date	0.01	<0.00	4.91	**<0.001**
(d) Hatching success (binomial GLM)				
Intercept	1.25	0.85	1.47	0.141
Site	−0.55	0.23	−2.36	**0.018**
(e) Fledging success (binomial GLM)				
Intercept	11.43	2.03	5.62	<0.001
Site	−5.95	1.02	−5.85	**<0.001**
(f) Number of fledglings (poisson GLM)				
Intercept	2.68	0.15	17.99	<0.001
Site	−1.01	0.10	−10.14	**<0.001**
(g) Nestling body mass at day 13 (LMM)				
Intercept	12.11	0.35	34.26	<0.001
Site	−0.91	0.20	−4.49	**<0.001**
(f) Adult body mass (LMM)				
Intercept	11.27	0.48	23.22	<0.001

Lay date, clutch size, hatch date and number of fledglings were modelled with a Poisson error structure, hatching success (number of hatchlings over laid eggs) and fledging success (number of fledglings over number of hatchlings), were modelled with a binomial error structure, while adult and nestling body mass were modelled with a Gaussian error structure in LMMs with nestbox included as random factor. Site was included in all models as main fixed effect, whereas date of first egg of each nest was included as covariate to control for seasonal variation in reproductive timing and body mass (except for the model testing for variation in lay date across sites). For adult body mass, sex was also included as fixed factor. These were dropped if not significant via backward selection (only significant variables are shown).

Body mass at day 13 was significantly lower in both suburban (mean ± s.e.m = 9.67 ± 0.26, P < 0.001) and urban nestlings (9.98 ± 0.37, P = 0.002) compared to the forest nestlings (11.36 ± 0.18; for details, see Tables 1, 2 and 3), whereas no significant difference was found between the suburban and urban nestlings (P = 0.755, Table 1). Conversely, adult body mass was not at all affected by site (P = 0.811, Tables 1, 2 and 3).

### Food availability: arthropod sampling

**Figure 1:**
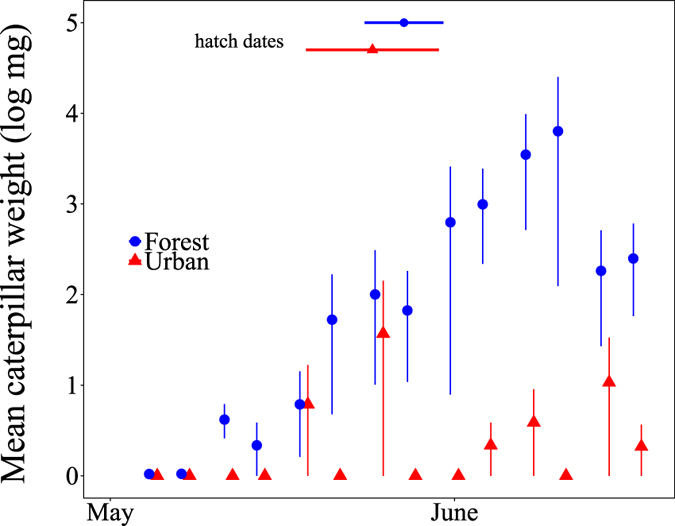
Caterpillar weight throughout the breeding season. Symbols depict mean ± s.e.m (log transformed) of dry weight of all caterpillars sampled from five different trees at each time point at each site; site is depicted by red (urban) or blue (forest) colours. Horizontal red and blue dashed lines represent hatch dates (mean ± s.e.m) for broods in the urban and forest site, respectively.

The total number of caterpillars as well as the mean weight of the caterpillars collected at each sampling session throughout the breeding season was considerably lower in the urban compared to the forest site (P < 0.001 for both variables, Tables 1, 2 and 4, Fig. 1). In particular, caterpillar weight showed a marked peak in the forest habitat, whereas the peak in the urban site was lower and less pronounced (Fig. 1). The two sites also varied considerably in the diversity and abundance of arthropods sampled (Table S2). Arthropod diversity was not different between the forest and urban samples at the beginning of spring, but while diversity increased with date in the forest, the opposite was true in the urban site (interaction site*date: P = 0.015, Table S3 and Fig. S3). The overall abundance of insects in the city trees generally outnumbered that in forest trees (total urban arthropods = 1553, total forest arthropods = 686: Table S2). Our model indicated a tendency for a significant interaction site between site and date (P = 0.080). Indeed, while there was no apparent difference in abundance between sites early in the season, from late May onward more arthropods were present in the urban than rural samples (Fig. S3). However, the great majority of these late urban specimens were aphids (Order: Hemiptera), which were not detected in the forest site (Table S2). When these samples were excluded from the model, neither the interaction between site and date, nor the main linear effects of site and date were found to significantly affect arthropod abundance (P > 0.1 in all cases). In addition, we also found differences between tree species: birches and oak samples contained more arthropods than samples from beech trees (Table S3).

**Table 4:** Summary of model testing for differences in caterpillar numbers and weight across sites and day of the breeding season, after backward selection.

Response					
Explanatory variable	Estimate	Std. Error	df	t value	P value
(a) Amount of caterpillars (log)					
Intercept	0.31	0.17	53	1.84	0.071
Site	−0.60	0.07	132	−7.90	**<0.001**
Day	0.01	0.01	132	2.34	**0.020**
(b) Caterpillar weight (log)					
Intercept	−0.02	0.01	130	−2.16	0.032
Site	0.02	0.01	130	1.74	0.084
Day	0.001	0.0002	130	3.54	**<0.001**
Site * Day	−0.001	0.0002	130	−2.76	**0.007**

Response variables were log-transformed to achieve normality of residuals. Initial explanatory variables were site, day and site*day interaction, and were dropped if not significant via backward selection (only significant variables are shown). Tree ID was always included as random factor.

### Diet provisioned to nestlings

**Figure 2:**
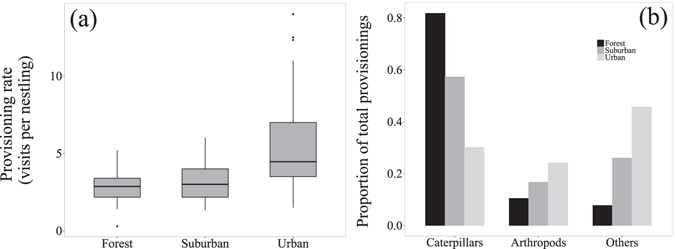
Provisioning of nestlings. (**a**) Provisioning rates of parents to offspring across the three study sites per 30 min. Rates are expressed per nestling, that is, number of visits per brood/brood size. (**b**) Proportions of total provisioned items for different groups of dietary components at each of the three study sites. Data for both panels were obtained from a total of 2 h of video footage combined from 4 different 30-min intervals recorded on the same day.

Birds at the urban site provisioned each nestling almost twice as often as birds in the forest (P = 0.007, Tables 1, 2 and 5; Fig. 2a), while provisioning rate did not differ between other combinations of sites (Tables 1, 2 and 5, Fig. 2a). The opposite pattern was detected for the total number of visits per nest: overall, urban parents provisioned their clutches less often than parents in the forest, although this was not significant (P = 0.096, Tables 1, 2 and 5). In addition, across all sites, both provisioning rate and total visits per nests were higher in the morning than during any other time of day (P < 0.001 for both variables, Table 5).

**Table 5:** Results from LMMs investigating how provisioning rates and dietary sources fed to nestlings varied across the three study sites.

Response variable					
Explanatory variable	Estimate	Std. Error	df	t value	P value
(a) Visits per nestling (log)					
Intercept	1.35	0.18	16.7	7.41	<0.001
Site	0.25	0.08	14.9	3.05	**0.008**
Time of day	−0.12	0.02	169.9	−6.75	**<0.001**
(b) Visits per nest					
Intercept	28.34	3.43	16.6	8.27	<0.001
Site	−3.29	1.52	15.0	−2.16	**0.047**
Time of day	−2.12	0.31	169.7	−6.74	**<0.001**
(c) Mean volume of caterpillars (log)					
Intercept	5.23	0.30	14.8	17.57	<0.001
Site	−0.33	0.14	14.9	−2.43	**0.028**
(d) Amount of caterpillars					
Intercept	24.59	2.43	16.4	10.11	<0.001
Site	−6.51	1.08	15.0	−6.01	**<0.001**
Time of day	−0.64	0.21	169.6	−3.07	**0.003**
(e) Amount of other arthropods (log)					
Intercept	1.16	0.38	25.6	3.08	<0.001
Time of day	−0.20	0.08	46.7	−2.63	**<0.011**
(f) Amount of unidentified items					
Intercept	1.60	1.23	27.4	1.30	0.206
Site	1.97	0.47	13.8	4.23	**0.001**
Time of day	−0.74	0.28	46.1	−2.63	**0.012**

In all models, site, time of day and date were included as fixed effects, and dropped if not significant via backward selection (only significant variables are shown). Nest-box (brood) was included as random factor. We indicate in brackets whether the response variable was log-transformed.

The diet fed to offspring varied considerably across sites (Tables 1, 2 and 5; Fig. 2b). The proportion of caterpillars provisioned over 30-min periods strongly decreased from the forest (82%, N = 462) to the suburban (54%, N = 67) and urban site (27%, N = 101), and these differences were statistically significant (Table 1). Similarly, the average volume of each caterpillar provisioned to the nestlings was doubled in the forest (mean ± s.d. = 134.12 ± 20.88 mm^3^) compared to the urban site (mean ± s.d. = 69.49 ± 12.54 mm^3^, P = 0.049, Tables 1, 2 and 5). No significant differences were found between the suburban and the other two sites for both volume and number of caterpillars fed to the broods (Table 1). Conversely, the proportion of other arthropods and unidentified items increased from the forest to the urban site (Tables 1, 2 and 5; Fig. 2b). However, only the difference in the number of unidentified items between the forest and urban site was statistically significant (P < 0.001, Table 1).

### Stable Isotope Analysis

**Figure 3:**
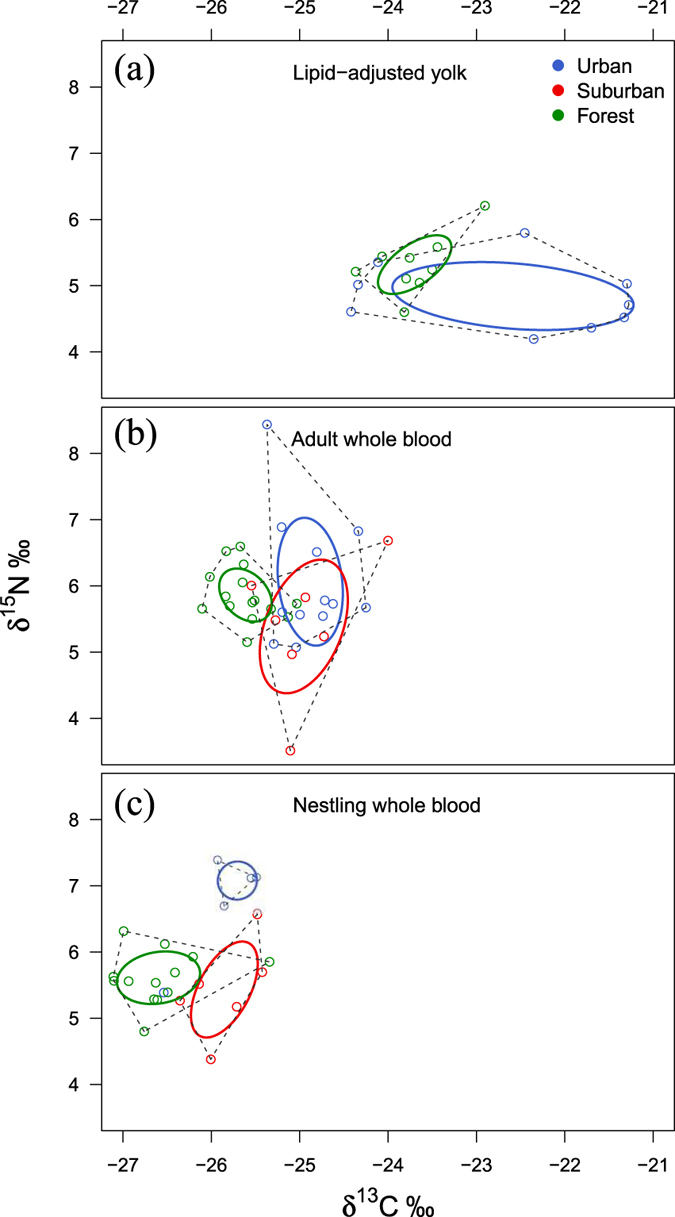
Isotopic niches extending across δ^13^C values (x-axis) and δ^15^N (y-axis) for tissues of blue tits from different sites. Isotopic niches for (**a**) yolk obtained from blue tit eggs, and for whole blood samples of (**b**) adults and (**c**) nestlings from the three study sites. Each dot represents one egg (**a**), one adult blood sample (**b**), and one blood sample pooled from 2–4 nestling siblings (**c**). The broken line represents total convex hull, and the solid ovals are ellipses that include approximately 40% of individuals within a group, which represents the breadth of the isotopic niche in each site.

In the egg yolk, both δ^13^C and δ^15^N were weakly but significantly different between the forest and the urban site (P = 0.031 and P = 0.050, respectively, Tables 1, 2 and 6; Fig. 3a). Specifically, δ^13^C was higher in the urban than the forest site, whereas the opposite held true for δ^15^N. For whole blood of adults, δ^13^C values in urban and suburban sites did not differ from each other (P = 0.959, Table 1), but they were both significantly higher than in the forest (P = 0.001 and P < 0.001, respectively, Tables 1, 2 and 6; Fig. 3b), Conversely, δ^15^N values did not differ across sites. In the nestlings, both δ^13^C and δ^15^N values of whole blood differed substantially across sites (both P < 0.001, Tables 1, 2 and 6; Fig. 3c). Values of both isotopes were higher in the urban compared to the forest site, and δ^15^N values were also higher in the urban compared to the suburban site (P = 0.001, Table 1).

**Table 6:** Results from LM analysis of stable carbon and nitrogen isotope signatures (δ^13^C and δ^15^N), after backward selection.

Tissue	Response variable				
	Explanatory variable	Estimate	Std. Error	t value	P value
Egg yolk	δ^13^C				
	Intercept	−23.70	0.33	−71.25	<0.001
	Site	1.11	0.47	2.36	0.031
	δ^15^N				
	Intercept	5.32	0.16	33.63	<0.001
	Site	−0.47	0.22	−2.12	0.050
Adult blood	δ^13^C				
	Intercept	−25.90	0.16	−164.18	<0.001
	Site	0.36	0.08	4.79	<0.001
	δ^15^N				
	Intercept	5.66	0.33	17.15	<0.001
Nestling blood	δ^13^C				
	Intercept	−26.94	0.22	−124.54	<0.001
	Site	0.41	0.11	3.70	0.001
	δ^15^N				
	Intercept	4.94	0.30	16.74	<0.001
	Site	0.54	0.15	3.58	0.001

Sampled tissues were eggs, and whole blood of adult and nestling blue tits. Initial explanatory variables were always site and date, and also sex for adult samples, and were dropped if not significant via backward selection (only significant variables are shown).

In all the three examined tissues, there was little or no overlap between the isotopic niches of the forest and the suburban/urban samples (Fig. 3). The sizes of the niches also differed: eggs and adult samples of urban and suburban birds had much larger niches than those from the forest (Table S4; Fig. 3). For nestling samples (Fig. 3c), the isotopic niche of the forest site was larger than that of the urban site (SEAc = 0.618 vs 0.366, Table S4; Fig. 3). However, when chicks originating from forest eggs were added to the analysis, the urban nestling niche became twice as large as that of the forest and extended towards the forest nestling niche (SEAc = 1.271 vs 0.618, Table S4; Fig. S4).

### Relationships between diet and breeding success

**Figure 4:**
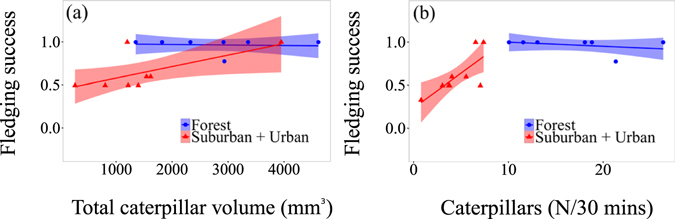
Fledging success of blue tits predicted from caterpillar provisioning. (**a**) Relationship of total volume of caterpillars fed to a brood (x-axis) and fledging success (number of fledglings divided by number of hatchlings, y-axis). Data for the urban and suburban site were pooled in a single “suburban + urban” variable because of low sample size due to high nestling mortality at these two sites. (**b**) Relationship between number of caterpillars fed to a brood over 30 min and its fledging success. In this case a significant interaction site*caterpillars was found, indicating a positive relationship only for the suburban + urban broods, whereas the fledging success of the forest broods was weakly affected by the number of caterpillars fed. Blue colour and dots represent forest, red colour and triangles represent suburban + urban birds. Each symbol represents a single nest. Lines represent estimates from linear regression models, and shaded areas s.e.

Fledging success increased with both the total volume and total number of caterpillars provisioned to a brood (Fig. 4). The total volume of caterpillars provisioned significantly predicted fledging success (t = 5.2, P < 0.001; Fig. 4a) independently of site, as the slopes for both the forest and the suburban + urban site (pooled to increase sample size) were similar (slope forest = 0.0001; slope urban + suburban = 0.00013). Conversely, we found a significant interaction between site and total amount of caterpillars in predicting fledging success (t = 4.4, P < 0.001; Fig. 4b). The effect of increasing numbers of caterpillars on fledging success was positive in the suburban + urban site (slope = 0.042), whereas no effect was evident in the forest site (slope = −0.003).

## Discussion

Here we show that in an urban site close to the city centre of Glasgow, breeding blue tits lack the optimal food source, caterpillars, to successfully raise their offspring.

### Available and Provisioned food

Overall, caterpillar availability was much lower in our urban compared to the forest site (Fig. 1), confirming findings from other studies^23, 38^. Differing habitat structures may contribute to explaining lower caterpillar availability. Tree densities in the surroundings of the nestboxes were much lower at the urban compared to rural sites (H. McDevitt *et al*., unpubl. data). The composition of plant species also differed between sites. In the urban park site, many trees were exotic species, which are known to reduce abundance and diversity of arthropods^39^, particularly lepidopteran larvae^40, 41^. Negative effects could be exacerbated by fragmentation and disturbance of lepidopterans, which may also play a role in regulating healthy population sizes^42^. The detected differences in caterpillar availability are expected to influence dietary provisioning, which largely depend on the immediate surrounding environment^31^. Nest video recordings and stable isotope analysis confirmed that the differences in food availability were indeed associated with differences in nestling diet.

Nest video recordings revealed that blue tit nestlings in the forest site were fed almost exclusively caterpillars, whereas in the suburban and especially the urban site, they also received other arthropods and unidentified items, resulting in broader dietary input. Although we could not identify with absolute confidence the provisioning of anthropogenic items from our video-recordings, field observations and anecdotal evidence strongly suggest so. Some parents trapped in nestboxes carried fat-ball (Fig. S7), and at a nearby site in Scotland, peanuts were found lodged in gullets of dead nestling (Fig. S8; Gerald Lincoln, pers. observation). Cowie and Hinsley^43^ also detailed for great tit nestlings that processing of human-provisioned items may be difficult for chicks. In addition, low quality of human-provisioned food may have also reduced breeding success^7^. Anthropogenic food supplies are often rich in calories but poor in proteins and vitamins. Such differences could also extend to natural diets from urban compared to forest sites. For example, urban caterpillars had low carotenoid content^26^, possibly due to low carotenoid content of inner-city trees^44^, and had also higher concentrations of pro-inflammatory fatty acids^45^.

### Assimilated diet

The shifts in diets between sites observed from camera recordings were confirmed by our stable isotope analysis. Our results indicate that in urban adults, eggs and nestlings, diets overlapped very little between the forest and the two sites in Glasgow. δ^13^C values obtained from the suburban and urban sites were similar to each other, and consistently higher than in the forest for all tissues analysed. Thus, it is likely that the carbon pools differed considerably between urban and rural sites. The trend towards higher δ^13^C signatures in urban areas has been recorded previously in other urban species^9, 46, 47^, and is suggested to be linked to the abundance of products with higher proportions of plant materials using the C4 photosynthetic pathway in human diet. In addition, higher δ^13^C values can also reflect differences in tree composition between different sites. Indeed, a recent study on great tits nestlings has shown that higher δ^13^C signatures were negatively and tightly correlated to the proportion of *Quercus* spp in the surrounding of the nest^37^. In accordance with this result, at our urban site there are comparatively fewer oaks than in the forest (H. McDevitt *et al*., unpubl. data). δ^15^N values were mostly similar across sites, except for nestlings in the urban site which showed much higher values than in the other two sites. An increase in δ^15^N has not been observed in previous studies on urbanised ants^9^ and coyotes^7^, but was seen in black bears feeding on anthropogenic sources^48^. Higher δ^15^N may suggest that urban parents were provisioning nestlings with arthropods at a higher trophic level. Indeed, parents appeared to provision more ladybirds and spiders at the urban than at the other sites, although the availability of insectivorous arthropods at our urban site was actually lower than that at the rural site. Alternatively or in addition, higher δ^15^N values in the urban site could be driven by anthropogenic food provided at bird feeders. For instance, fat balls are rich in animal products and may have contributed to the higher δ^15^N found in urban nestlings. Preliminary stable isotope analysis of common food provided at feeders confirmed higher δ^15^N for fat balls, multi-seed mixes and sunflower seeds compared to caterpillars collected in both the urban and forest sites (Table S5). In addition, and according to our predictions, the isotopic niches we calculated for adult birds and their eggs were considerably broader in the urban site than in the forest, indicating utilisation of a wider variety of dietary sources by urban adult birds. However, for nestlings the isotopic niche was smaller for city than that for forest birds, which might be due to low sample sizes. When we included data from city nestlings hatched from cross-fostered forest eggs, the isotopic niche became larger in the city than in the forest (Fig. S4). However, the increased niche breadth may reflect an isotopic signature in nestlings composed of post-hatching diet and maternal investment. This interpretation is suggested by the greater proximity of the isotopic values of city-reared chicks hatched from forest eggs to those of forest chicks. Finally, the different isotopic signatures in the tissue of urban and rural birds could also reflect habitat-specific isotopic signatures, rather than dietary shifts of birds^9^. In order to exclude this possibility, future studies should analyse isotopes from urban and forest plant species.

### Fledging success and body mass

Our study showed substantially reduced fledging success and body mass with increasing urbanisation. The observation that urban parents provisioned each nestling almost twice as often as forest parents suggests that poor diet, rather than reduced parental investment, underlied their compromised breeding performance. Our findings that the total volume and to some extent the total number of caterpillars fed to a brood predicted fledging success match existing data on blue tits^49^. Interestingly, the relationship between the number of caterpillars provided to a brood and its fledging success seems to be dependent on the habitat considered: higher caterpillar numbers were highly correlated with fledging success at the urban site, but not at the forest site, where parents provisioned on average more with no further gain in fledging success (Fig. 4b). In an investigation into caterpillar abundance and foraging behaviour across different quality habitat types, Tremblay *et al*.^31^ concluded that only if caterpillar abundance falls below a certain threshold, it explains fluctuations in fledgling mass and success. Figure 4 suggests that blue tits at our urban site may operate below this proposed threshold.

In stark contrast to post-natal traits such as nestling body mass and fledging success, all major pre-natal life-history traits (lay date, clutch size, incubation period, hatching success) differed little across sites, confirming that environmental effects during rather than before the nestling period are likely responsible for the reduced breeding performance of urban birds.

### Caveats and conclusions

We acknowledge that our sample sizes are low across our different sites, but despite low power the effect sizes are in most cases large. In addition, although our study focused on only one urban gradient, most behavioural and fitness traits were intermediate in the suburban site, which reinforces the idea that urbanisation *per se* is responsible for the effects we found. However, our data come from a single year which was exceptionally poor in terms of blue tit breeding success. We have recorded life-history data at our sites for the three years of 2014 until 2016. In our study year 2015, brood size (~6 nestlings in the forest) was considerably lower than both the usual average for blue tits^6, 50^ and our own data from 2014 and 2016 (~10 nestlings in the forest). Reproductive success was also strongly reduced at all three sites compared to 2014 and 2016, but especially so at the urban sites (Fig. S2). Thus, our data suggest that conditions for breeding in 2015 were suboptimal, but blue tits were better able to cope in the forest compared to the urban habitat. ﻿In accordance to this evidence, a complementary study done in the same sites in 2015, but on different individuals, revealed that adult urban blue tits showed higher levels of expression of inflammatory markers compared to forest birds﻿﻿﻿^51^, confirming the suboptimal breeding conditions of the urban habitat.﻿

In contrast to many other studies, our research has taken a decidedly comprehensive approach to avian urban ecology, which is however traded off against the number of sites that can be studied at such depth. Hence, insights from our study need to be considered tentative until further research will confirm they can be generalised to more urban and forest sites. While we acknowledge that our design cannot rule out effects of inter-site variation, rather than urbanisation *per se*, we believe that studies with emphasis on comprehensiveness and replication, respectively, both play important and complementary roles in ecology. Our study contributes to an increasing understanding of the constraints associated with city life for breeding birds. Although reduced reproductive success is common in urban birds, it remains an important research objective to obtain long-term data on bird productivity as well as food availability along urban gradients. Such long-term data will enable us to elucidate the frequency and magnitude of crashes in productivity in urban bird populations, like those observed at our sites, and estimate expected implications for urban population dynamics^27^. Our findings provide food for thought for urban ecologists and call for further investigation. Understanding the drivers of differences in food availability in urban areas and their consequences for the health of urban populations could also make a real difference for the developing plans that aim to conserve urban biodiversity. The findings from our study suggest that a primary objective should be to increase the birds’ provisioning of lepidopteran larvae, for example by prioritising native plant species that are hosts to lepidopterans, and by circumspect use of bird feeding during the breeding season.

## Materials and Methods

### Ethical statement

All bird sampling was conducted following the directions and legislations of UK Home Office. The experimental protocols were approved by the UK Home Office (blood sampling: project licence 70/7899 to BH, and personal licences to DMD and BH), the Scottish Natural Heritage (Pit-tags: Permit 52463 to BH) and the British Trust for Ornithology (Aluminum rings: Scientific C and T licences to BH and DMD, respectively).

#### Study sites and bird sampling

We conducted this study across three sites chosen to represent an urban to rural gradient between Glasgow and a native oak woodland forest in Loch Lomond National Park, 40 km north of Glasgow, UK (Table S1), from beginning of April to end of June 2015. Our focal analyses concentrated on the forest and urban endpoints, while the suburban site was used to test for specific intermediate values along a gradient. At least 40 nest-boxes (Woodcrete, Schwegler, Germany; dimension = 260H × 170W × 180D cm; hole diameter = 32 mm) were installed at each site, at approximately 50 m from each other in variable cardinal directions (Table S1).

Nest-boxes were monitored weekly during the nest-building and incubation phases, and all reproductive activities were recorded. When the expected hatching date was approaching, we monitored nests every other day to identify hatching dates and hatching success. Broods were then left undisturbed until day 13, when chicks were weighed, ringed and blood samples obtained. During the clean out of boxes we checked for any remaining dead nestlings. Based on these data, we defined hatching success as the number of chicks hatched divided by clutch size, and fledging success as the number of chicks counted on post-hatching day 13, minus any dead young found during clean-out, divided by number of hatchlings.

We present breeding and nestling mass data from all occupied nest-boxes in the three sampled sites (Forest = 35, Suburban = 27, Urban = 29). All broods studied are first broods (blue tits very rarely have second broods in Scotland). We then selected a subset of blue tit nests at each site to be studied in detail for both provisioning behaviour and stable isotope analysis. We initially aimed to randomly sample at least 10 broods in each site. However, mortality was very high due to the poor weather conditions throughout the season, thus especially in the urban and suburban sites we had to limit our sampling to the broods that were available and contained at least two nestlings.

#### Arthropod sampling

As blue tits are primarily arboreal feeders we monitored differences in caterpillar and other potential arthropod prey types throughout the breeding season at the urban and forest endpoints, using a tree beating technique52. Three native tree species were selected in each site (Quercus spp N = 2, Betula spp. N = 2, Fagus spp. N = 1) seeking a balance between species of importance for arthropod communities (oak, birches), and representing the dominant tree species of both sites (mainly oak in the forest, and a mix of oak, birch, beech and ornamental trees in the urban parks, Table S1). We repeated the tree beating procedure twice a week from the end of April until mid-June (total trees sampled = 10, replicated measurements per tree = 14). Four branches per tree were beat on a rotational basis to minimise sampling effects. Arthropod samples were counted and identified to order level (total arthropod samples identified = 2239). All caterpillars (Order: Lepidoptera) were retained, desiccated in a drying oven at 50 °C for 24 hours, and then weighed (mg) to represent caterpillar biomass. We derived two measures of caterpillar abundances: mean dry weight and total number of caterpillars, per sample session for each site. A subsample of caterpillars and spiders (Order: Araneae) were retained and dried in the same conditions to be used in stable isotope analysis.

#### Parental provisioning behaviour and diet provisioned

In order to record differences in diet provisioned to nestlings, we installed video-recording systems (Forest N = 7, Suburban N = 3, Urban N = 7) in the back of nest boxes facing their entrances. These systems were custom-made by the University of Glasgow’s Bioelectronics Unit (sample pictures are shown in Figs S5, S6 and S7). Systems were deployed the evening of day 11 of the nestling period, and recordings made on the undisturbed day 12 were used for analysis. All recordings showed that parents started feeding at a normal pace in the morning, suggesting that they were not affected by the presence of the cameras. Males and females could not be discriminated from our video-recordings. Four half hour periods throughout the day (07:00 “morning”, 11:00 “midday”, 16:00 “afternoon”, 20:00 “evening”) were analysed for each box recorded using VideoLAN VLC Media Player (Paris, France). For each visit the time was recorded along with the type of prey, classified in three categories: caterpillar, other arthropods, and unidentified food items which could not be visually verified with complete confidence (22%, N = 225). A volume index was calculated for > 95% (N = 630) of caterpillars provisioned to nestlings using the formula (π/4)*L*W2, where L is the total length and W the mean width relative to the diameter of the nest hole (32 mm)30. Total number of visits recorded over the half hour period provided a record of provisioning rates per brood. This was divided by brood size measured on the day of deployment of the camera to estimate the number of visits each nestling received in that half hour period.

#### Assimilated diet

Sample collection and preparation. For a subset of nests, one egg (Forest = 9, Urban = 9) was removed during the laying phase and a surplus egg from a non-focal nest from the same site was introduced in attempt to minimise disruption and maintain original brood traits. Eggs were divided into yolk, where isotopic values reflect diet over eight days prior to laying^53^, and albumen and stored in 1.5 ml Eppendorf tubes in a freezer at −80 °C. After thawing yolk and albumen were oven-dried at 60 °C for 48 hours and homogenized using a mortar and pestle. Due to the high lipid content of yolk a lipid extraction was performed by washing a subsample of the yolks with a 1:1 chloroform and methanol solution three or more times, and then rinsed with distilled water and dried at 40 °C for another 24 hours.

Adult birds (Total [males, females]: Forest = 15 [5, 15], Suburban = 7 [1, 6], Urban = 12 [4, 8]) were caught while provisioning in the nest-boxes and bled on day 11 of the nestling period, while nestlings (Forest = 13 nests, Suburban = 6 nests, Urban = 7 nests) were bled at age 13 days. Blood (50–70 μl), which integrates dietary information over several days to a few weeks^35, 54^, was collected from the brachial vein into heparin capillary, transferred to a glass vial and then stored at −80 °C until required. To reduce the amount of blood taken from nestlings we pooled samples from several individuals, obtaining a single, pooled sample per brood (N = 2–4 nestlings per brood). Nestlings were not sexed, and we avoided sampling nestlings with body mass < 9 g to avoid imposing further stress on them. Directly after thawing, blood samples were oven-dried at 60 °C for 48 hours and were then powdered.

Stable isotope analysis. Carbon and nitrogen stable isotope assays were performed on dry and homogenized samples of approximately 0.7 mg that were weighed into tin cups and combusted at approximately 940 °C in an Elementar PYRO Cube. CO_2_ and N_2_ gases emitted were then analysed in an interfaced Thermo Finnigan Scientific DELTA PLUS XP isotope ratio mass spectrometer with every 10 unknowns separated by two of three laboratory standards (Fluka gelatin, Sigma alanine or Sigma glycine (Sigma-Aldrich Company Ltd, Gillingham, UK)). All stable isotope ratios are expressed in per mil (‰) using the δ notation: δ X = [(R_sample_/R_standard_) − 1], where X is the ^15^N or ^13^C and R is the corresponding ratio of heavy/light (^13^C/^12^C or ^15^N/^14^N) isotope. R_standard_ is the ratio of the international references, which for carbon is Vienna Pee Dee belemnite (VPDB), and for nitrogen is AIR^55^. Analysis were conducted at the Scottish Universities Environmental Research Centre (SUERC) stable isotope laboratory, East Kilbride.

#### Statistical analysis

All analyses were conducted within the statistical environment R^56^, using functions in the basic version of the software or specific packages outlined below. When linear models (including mixed models) were used, the assumptions of normality of residuals and homogeneity of variance were checked by inspecting residuals plots. All linear mixed models (LMMs) were based in the package *lme4* ^57^. We always started by analysing full models that contained all biologically meaningful explanatory variables. We then performed a backward selection procedure^58, 59^ by eliminating non-significant variables based on their p values. These were calculated based on F tests (for LMs), z tests (GLMs), or t-tests using Satterthwaite approximation to degrees of freedom (LMMs), the latter available in the R package *lmertest* ^60^. Tukey’s post hoc tests were run to obtain p-values for each pairwise contrast between the three levels of the variable site (forest, suburban, urban), using the package *multcomp* ^61^.

Reproductive performance and body mass. We used generalised linear models (GLMs) or LMMs to analyse measures of reproductive performance and body mass. We modelled reproductive data in GLMs with either a Poisson error structure (for lay date, clutch size, hatch date and number of fledglings) or with a Binomial error structure (for hatching or fledging success) in independent GLMs. The latter was used because hatching and fledging success were calculated as proportion of chicks hatched or fledged from the initial number of eggs laid in a clutch. Nestling and adult body mass were modelled as response variables with a Gaussian error structure in two independent LMMs, with nestbox as random factor to control for non-independence of data points as sibling chicks as well as some adults (pairs) came from the same nestbox. Site was included in all models as main fixed effect, whereas date of first egg of each nest was included as covariate to control for seasonal variation in reproductive timing and body mass (except for the model testing for variation in lay date across sites). For adult body mass, sex was also included as fixed factor.

Arthropod sampling. For the analysis of caterpillar abundance and weight (both log-transformed), we used separate LMMs with site as fixed effect, and tree and day as random effects to correct for repeated measures. Arthropod diversity and total arthropod counts were analysed with a LMM and a Poisson GLMM, respectively, with site, day, tree species and the interaction of site and day as explanatory variables, and tree as random factor. We accounted for overdispersion in the GLM introducing an observation-level random effect. Diversity of arthropod communities present was calculated on total orders identified on each sampling day for each tree, using the Shannon’s index^62^.

Diet provisioned to nestlings. We used LMMs to investigate how the type and amount of prey provisioned differed across sites. The unit of analysis was the 30-min video-recording, replicated four times during the day. In seven independent models we used as response variables: number of visits per nestling, total number of visits per nest, the average volume of caterpillars fed to the nestlings, number of caterpillars, arthropods and unidentified items fed to nestlings. These variables were log-transformed when necessary to achieve normality of residuals and homogeneity of variance. In all models, site, time of day and date were included as fixed effects, whereas nest-box (brood) was included as random factor.

Stable Isotope Analysis. We compared the isotopic signatures of blue tit tissues across sites with linear models (LMs), using δ^13^C and δ^15^N as response variables, for each type of tissue tested (egg yolk, adult and nestling blood). Explanatory variables included site and date, and also sex for adult samples.

To compare the breadth of isotopic niche between different populations and tissues, we used the R function SIBER contained in the *SIAR* package^63, 64^. This function calculates standard ellipses to represent the isotopic niche width of 40% of typical individuals within the groups based on bivariate normal distributions. To control for small sample size, we used the corrected version of the standard ellipse area (SEA_c_), which is calculated from the total convex hull area.

In parallel with this study, we conducted a separate cross-fostering experiment investigating the effects of urbanisation on immune responses, using different blue tit nests at the same sites (Capilla-Lasheras et al, unpublished results). To increase sample sizes of stable isotope data, which had been low in the city due to high-mortality, we additionally analyzed blood samples from urban nestlings that had hatched from eggs originally laid in the forest (n = 3 nests). We present analyses that included these cross-fostered chicks as Supplements (Table S4; Fig. S4).

Relationships between diet and breeding success. The relationship between fitness parameters and diet was assessed with LMs. As dietary information we used the data collected from camera recordings rather than isotope signatures, because the latter can integrate other information than diet itself (see ref. 9 and discussion section). We modelled fledging success of a nest as response variable and included as fixed effects the number of caterpillars, other arthropods or unidentified items fed to the nestlings. As several broods were found dead in both the urban and suburban site during the last fledging check, sample size did not allow us to run the model separately for these two sites. Thus, we pooled them and named this new level of the factor site “suburban + urban”.

### Data accessibility

Data will be deposited in the Dryad Repository when the article will be accepted.

## Electronic supplementary material

Supplementary material
