# Supplementary material for: Integrated behavioural and stable isotope data reveal altered diet linked to low breeding success in urban-dwelling blue tits (Cyanistes caeruleus)
Source: Sci Rep. 2017 Jul 10;7:5014. doi: 10.1038/s41598-017-04575-y (PMC5503996; doi:10.1038/s41598-017-04575-y)

SUPPLEMENTARY MATERIALS FOR:

Integrated behavioural and stable isotope data reveal altered diet linked to low breeding success in urban-dwelling blue tits (*Cyanistes caeruleus*)

Christopher J. Pollock, Pablo Capilla-Lasheras, Rona McGill, Barbara Helm, Davide M. Dominoni

SUPPLEMENTARY DESCRIPTIVE TABLE

Table S1. Description of study sites and sampling conducted at each of them.

	**Cashel Forest** (56° 11' N, -4°57' W)	**Garscube** (55º 9’ N, -4º31’ W)	**Kelvingrove Park** (55°86’ N, -4°29’ W)
**Type**	Deciduous forest (forest extreme)	Suburban parkland (midpoint)	Inner-city parkland (urban extreme)
**Tree species composition**	Majority native species; oak (*Quercus* spp.), and birch (*Betula* spp.)	Open parkland, with introduced plant species alongside a good proportion of native oak and birch trees.	Small size parkland, embedded in dense urban matrix, with many introduced species and only sparse birch and oak trees.
**Total nest boxes (occupancy rate)**	94 (37%)	40 (67%)	60 (50%)
**Sample sizes**			
1) Life history data (number of boxes)	35	27	29
2) Invertebrate sampling (total samples)	70 (5 x 14)	0	70 (5 x 14)
3) Parental provisioning (number of nests)	7	3	7
4) Stable isotope analysis (number of egg, adult and nestling samples)	9, 15, 13	0, 7, 6	9, 12, 7

LIFE HISTORY AND BODY MASS DATA

Figure S1. Variation across the three study sites in life history variables: (a) clutch size, (b) hatching date (1 = April 1st), (c) hatching success (number of hatchlings/clutch size), (d) fledging success (number of fledglings/number of hatchlings). In panels (a) and (b) data are summarised as boxplots, whereas (c) and (d) show mean barplots with error bars representing s.e.m. Sample sizes: Forest = 35, Suburban = 27, Urban = 29 nests.

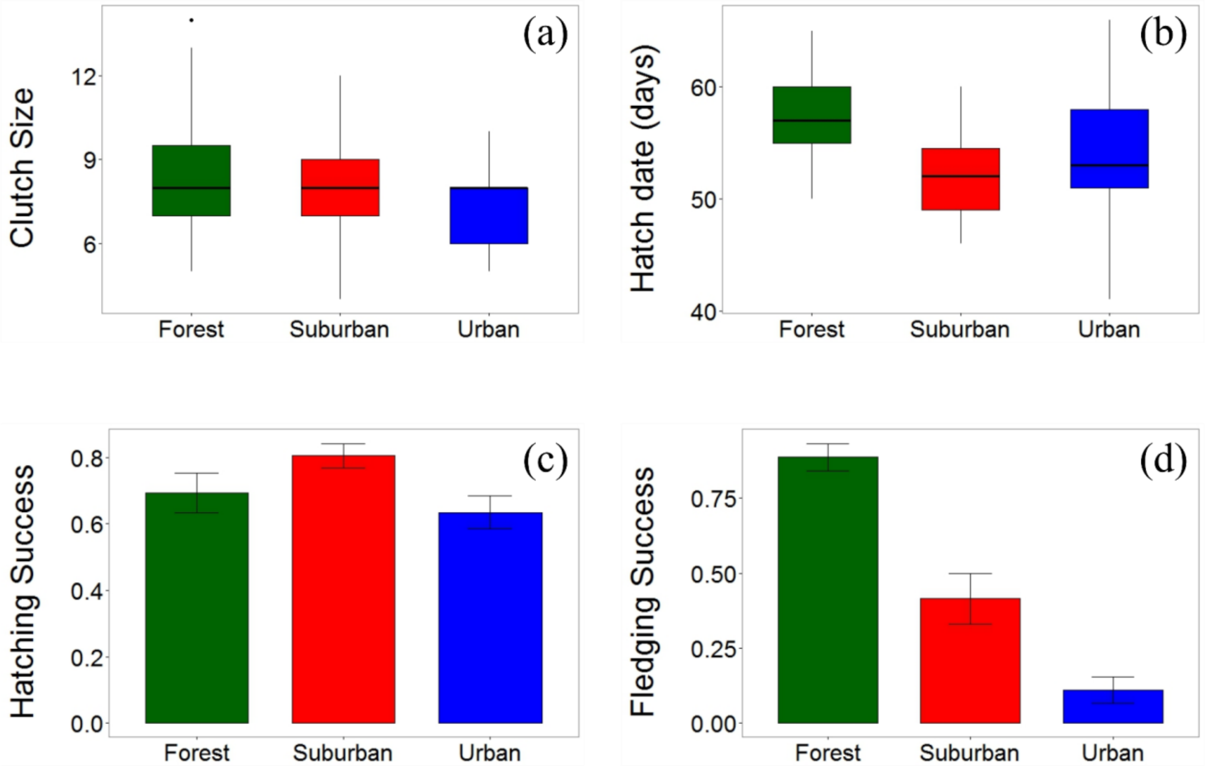

Figure S2. Interannual variation across the three study sites in fledging success. Full circles and error bars represent mean ± s.e.m. We ran a LMM as: fledging success ~ site+year+first egg date. Nestbox was included as random factor. All explanatory variables were highly significant ($P < 0.001$), and Tukey's post-hoc tests on the variable treatment revealed highly significant differences between urban-forest and suburban-forest sites ($P < 0.001$), but not between urban and suburban sites ($P = 0.58$).

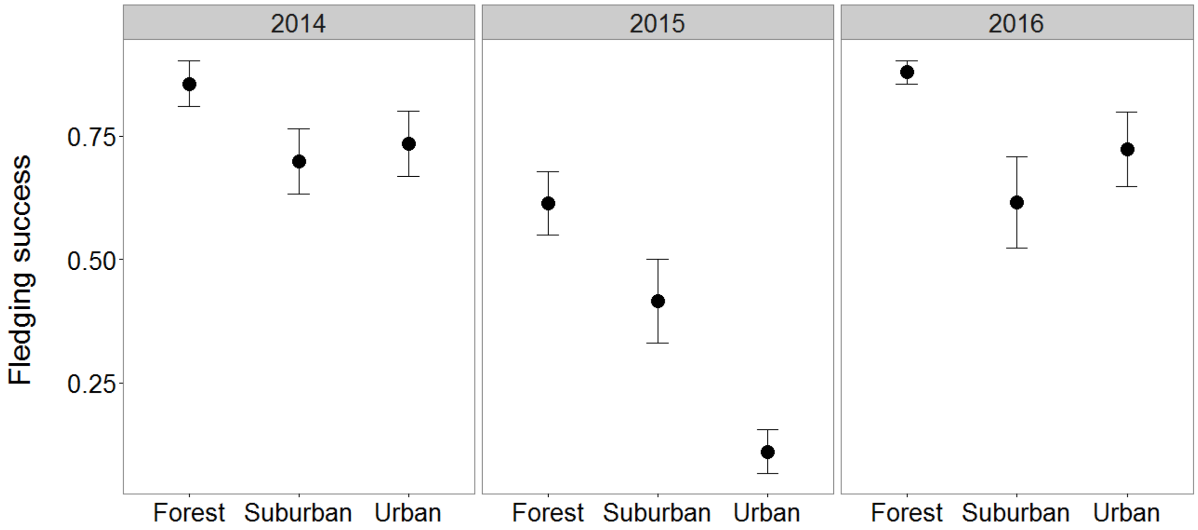

ARTHROPOD DATA: FOOD AVAILABILITY

Table S2. Arthropod abundance and diversity metrics based on tree-beating at the urban and forest sites. Results are total numbers per tree sampled throughout the bird breeding season.

	Urban					Forest				
**Tree**	Birch 1	Birch 2	Oak 1	Oak 2	Beech	Birch 1	Birch 2	Oak 1	Oak 2	Beech
**Samples**	13	13	13	13	13	13	13	13	13	13
Lepidoptera	0	3	2	2	0	39	6	31	10	7
Arachnida	9	8	7	3	2	24	65	61	39	21
Coleoptera	23	9	25	8	5	20	6	55	12	7
Hemiptera	324	221	564	65	91	12	4	29	5	1
Diptera	9	46	32	23	33	4	32	36	30	19
Other	19	6	2	9	3	24	17	43	16	11
**Total orders**	9	9	8	7	5	9	11	13	10	8
**Total Arthropods**	384	293	632	110	134	123	130	255	112	66
**Shannon Diversity:**	0.70	0.91	0.50	1.12	0.83	1.74	1.45	1.92	1.79	1.67

Table S3. Results from LMMs and GLMs investigating how Shannon diversity index and total arthropods varied between the urban and the forest site. Tree ID was always included as random factor. Reference level for the variable Site is rural, for Species it is beech.

**Response variable** / **Explanatory variable**	**Estimate**	**Std. Error**	**df**	**t or z value**	**P value**
a) Shannon diversity index (LMM)					
Intercept	0.82	0.24	103.6	3.43	< 0.001
Site	0.22	0.32	97.5	-0.69	0.491
Day	0.00	0.00	123.22	-1.09	0.276
Site*Day	-0.01	0.00	123.15	2.47	**0.015**
b) Total arthropods (Poisson GLM)					
Intercept	0.03	0.40	103.6	2.31	0.021
Site	0.47	0.26	97.5	1.94	0.066
Day	0.01	0.00	123.22	2.59	**0.010**
Species					
Birch	0.82	0.35	129	2.31	**0.021**
Oak	0.77	0.35	129	2.19	**0.029**

Figure S3. Variation in (a) arthropod diversity and (b) total arthropod counts between our urban and forest sites during the breeding season. Each point represents one tree sample obtained on one specific sampling day.

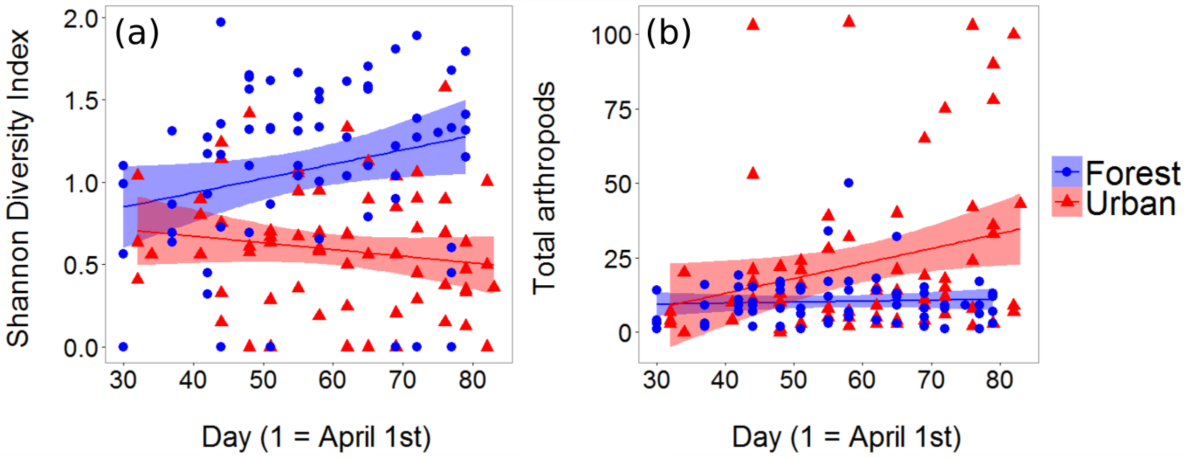

STABLE ISOTOPE DATA: ASSIMILATED DIET

Table S4. Isotopic niche values: TA = total area of convex hull, and SEAc = standard ellipse area corrected for sample size. Sampled tissues were a) eggs, and whole blood of b) adult and c) nestling blue tits. Values from urban nestlings were analysed including or excluding three nests whose eggs actually came from the forest as part of a separate cross-fostering (CF) experiment (see also Fig. S4).

Tissue / Site		TA	SEAc
a)	Egg yolk		
	Forest	0.740	0.515
	Urban	3.394	2.375
b)	Adult blood		
	Forest	0.893	0.373
	Suburban	2.076	1.706
	Urban	2.373	1.187
c)	Nestling blood		
	Forest	1.373	0.618
	Suburban	0.905	0.888
	Urban - including CF	1.594	1.271
	Urban - excluding CF	0.177	0.366

Table S5. Preliminary isotope values from potential food sources available to blue tits

Potential food sources	n	$\delta^{13}C$	s.d.	$\delta^{15}N$	s.d.
**Natural**					
Caterpillar - forest	3	-27.91	0.17	2.25	0.59
Caterpillar - urban	1	-29.09	NA	0.29	NA
Spider	3	-27.91	0.01	5.12	0.10
**Anthropogenic**					
Peanut	1	-24.38	NA	1.23	NA
"Mixed seed"	1	-23.36	NA	4.36	NA
Fat ball	2	-26.88	0.06	4.77	0.93
Sunflower seed	1	-26.69	NA	4.11	NA

Figure S4. Stable isotope niche plots for whole blood of blue tit nestlings, (a) excluding nestlings from eggs which originated from the forest; and (b) including them. Broken line represents total convex hull, and the solid ovals are ellipses that include approximately 40% of individuals within a group.

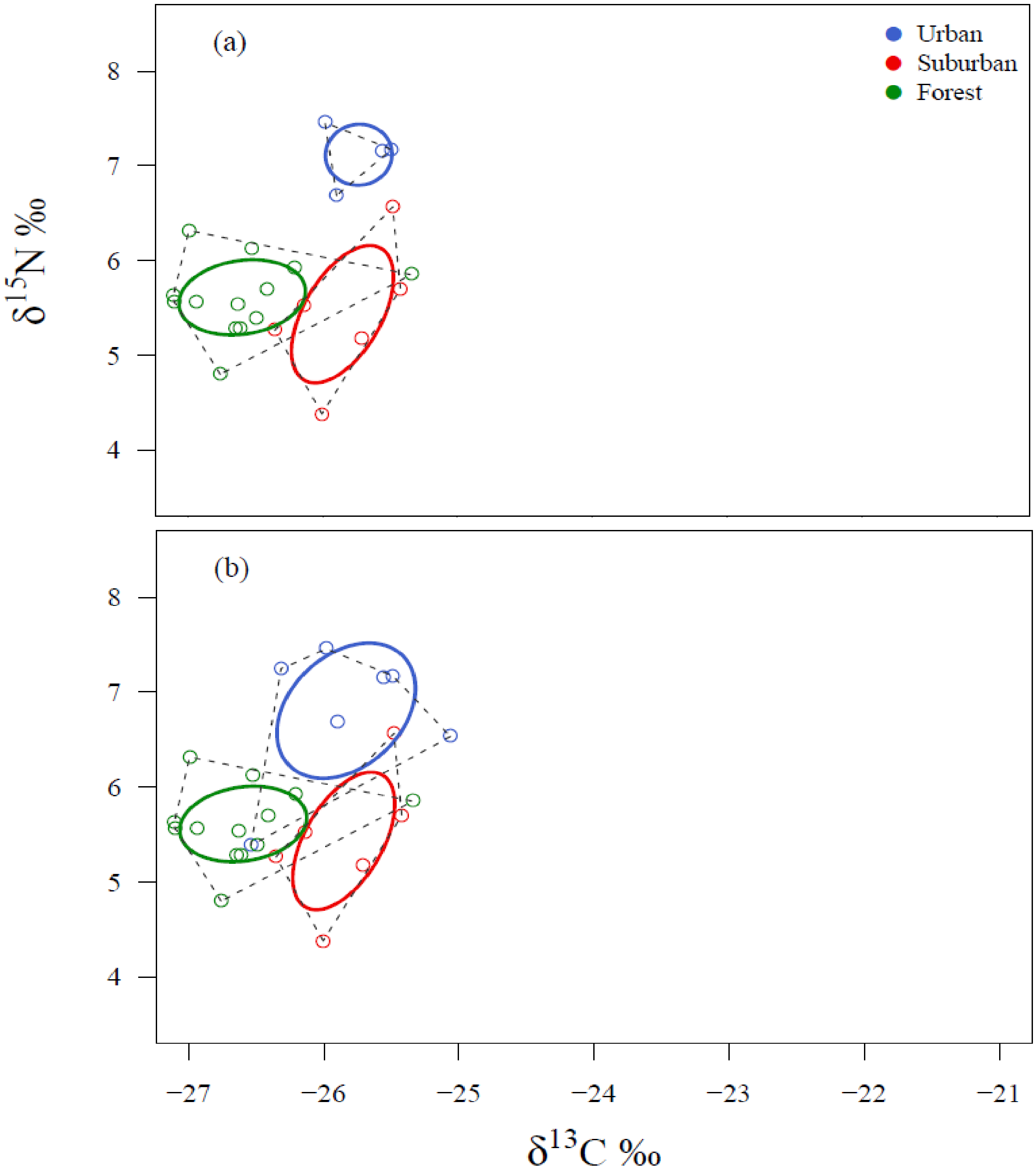

ADDITIONAL FIGURES

Figure S5. Caterpillar being brought to nestlings in an urban nest box.

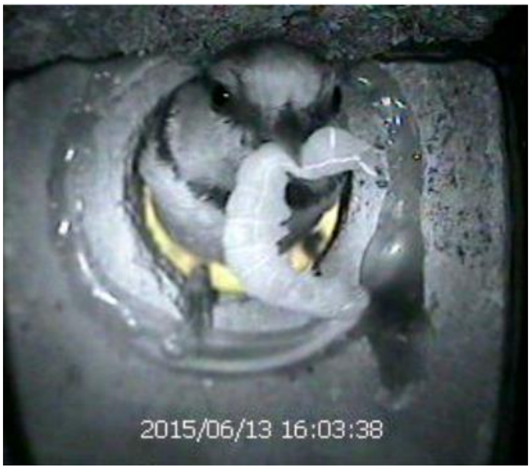

Figure S6. Ladybird being brought to nestlings in an urban nest box.

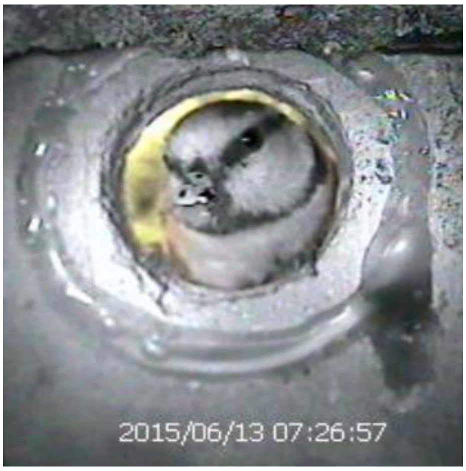

Figure S7. A non-invertebrate item, possibly of anthropogenic origin, being provisioned to nestlings in an urban nest box.

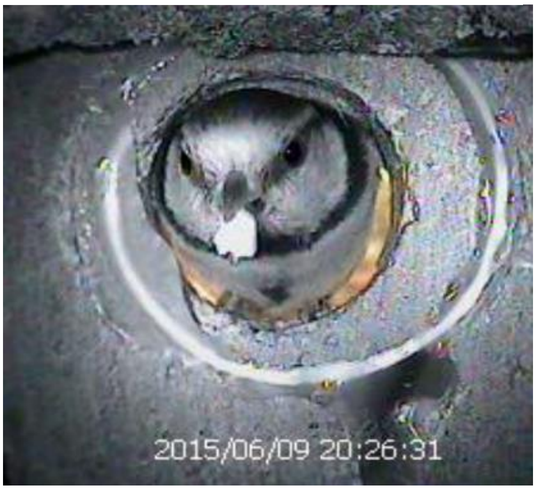

Figure S8. Nestling presumed to have died from starvation due to peanut pieces (bottom right corner) stuck in gullet. Photo: Gerald Lincoln.

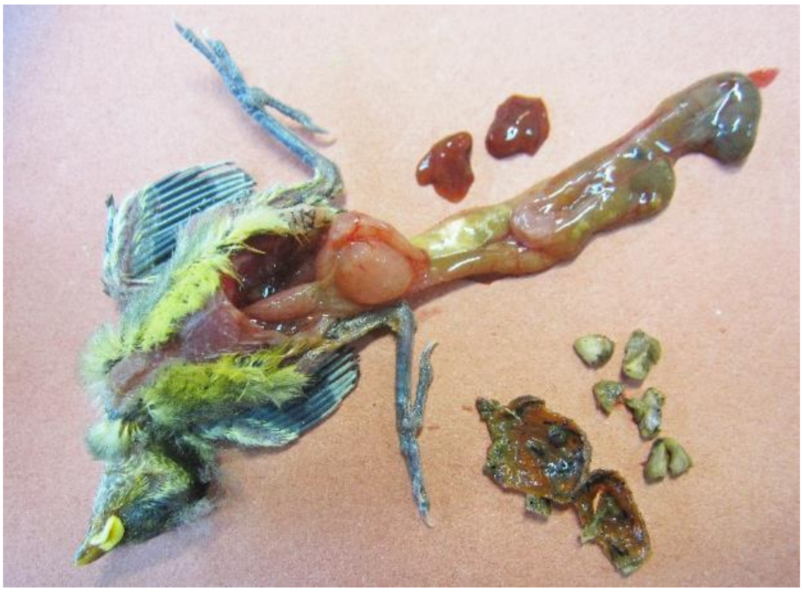

Supplement: Supplementary file 1 — Supplementary material [file 41598_2017_4575_MOESM1_ESM.pdf]
